# Among‐individual behavioural variation in the ornamental red cherry shrimp, *Neocaridina heteropoda*

**DOI:** 10.1002/ece3.11049

**Published:** 2024-02-22

**Authors:** Rosie Ann Rickward, Francesca Santostefano, Alastair James Wilson

**Affiliations:** ^1^ Centre for Ecology and Conservation University of Exeter Cornwall UK; ^2^ Département des Sciences Biologiques Université du Québec à Montréal Montréal Quebec Canada

**Keywords:** animal personality, behavioural syndrome, behavioural variation, boldness, cherry shrimp, decapod

## Abstract

Personality variation, defined as among‐individual differences in behaviour that are repeatable across time and context, is widely reported across animal taxa. From an evolutionary perspective, characterising the amount and structure of this variation is useful since differences among individuals are the raw material for adaptive behavioural evolution. However, behavioural variation among individuals also has implications for more applied areas of evolution and ecology—from invasion biology to ecotoxicology and selective breeding in captive systems. Here, we investigate the structure of personality variation in the red cherry shrimp, *Neocaridina heteropoda*, a popular ornamental species that is readily kept and bred under laboratory conditions and is emerging as a decapod crustacean model across these fields, but for which basic biological, ecological and behavioural data are limited. Using two assays and a repeated measures approach, we quantify behaviours putatively indicative of shy–bold variation and test for sexual dimorphism and/or size‐dependent behaviours (as predicted by some state‐dependent models of personality). We find moderate‐to‐high behavioural repeatabilities in most traits. Although strong individual‐level correlations across behaviours are consistent with a major personality axis underlying these observed traits, the multivariate structure of personality variation does not fully match a priori expectations of a shy–bold axis. This may reflect our ecological naivety with respect to what really constitutes bolder, more risk‐prone, behaviour in this species. We find no evidence for sexual dimorphism and only weak support for size‐dependent behaviour. Our study contributes to the growing literature describing behavioural variation in aquatic invertebrates. Furthermore, it lays a foundation for further studies harnessing the potential of this emerging model system. In particular, this existing behavioural variation could be functionally linked to life‐history traits and invasive success and serve as a target of artificial selection or bioassays. It thus holds significant promise in applied research across ecotoxicology, aquaculture and invasion biology.

## Graphical abstract

We investigate the structure, sex‐ and size‐dependence of personality variation in the red cherry shrimp, *Neocaridina heteropoda*, a popular ornamental species that is readily kept and bred under laboratory conditions and is emerging as a decapod crustacean model across subfields of ecology and evolution. Using a multivariate approach, we find individual behavioural differences that are repeatable across time and context, but not fully consistent with our a priori expectations of a shy–bold personality axis. We find some limited support for size‐personality association, but no evidence of sexual dimorphism.

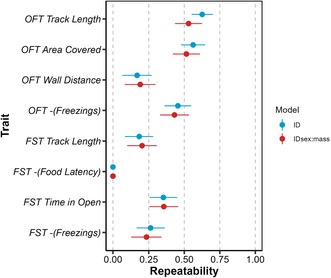

## 1 INTRODUCTION

Within populations, individual animals often exhibit behavioural differences that are consistent across time and context (Réale et al., 2007; Réale, Dingemanse, et al., 2010; Sih et al., 2004). Although this phenomenon, widely referred to as animal personality (Gosling, 2001), has been demonstrated across very diverse animal taxa (Bell et al., 2009), the empirical literature is dominated by vertebrate studies (Kralj‐Fišer & Schuett, 2014; Mather & Logue, 2013). In comparison, personality variation in invertebrates generally, and aquatic invertebrates in particular, has been less well studied (Gherardi et al., 2012). This omission matters because understanding personality variation is not only central to fundamental research on animal behaviour, but also increasingly relevant across more applied fields such as welfare (Prentice et al., 2022), ecotoxicology (Bertram et al., 2022; Ford et al., 2021) and invasion biology (Juette et al., 2014).

From a fundamental perspective, among‐individual variation in behaviour is widely assumed to have functional importance, impacting fitness via effects on survival and/or reproduction (Bertram et al., 2022; Briffa & Weiss, 2010; Moiron et al., 2020; Smith & Blumstein, 2008). While quantitative genetic studies have also shown that among‐individual differences are often heritable (Charmantier et al., 2014; Dochtermann et al., 2019; Stirling et al., 2002), understanding the evolutionary causes and consequences of this variation remains a challenge. Why does variation persist? Are among‐individual differences adaptive or do they reflect evolutionary constraints (e.g. trade‐offs)? Why can't all individuals adjust behaviour plastically to be optimal for the conditions they find themselves in? Hypothesised answers to these questions often invoke state‐dependence, predicting that personality will covary with other traits (e.g. metabolic rate, body size) or intrinsic variables (e.g. age, sex) as a consequence of feedback between behaviour and state (Biro & Stamps, 2008; Dingemanse & Wolf, 2010; Ferderer et al., 2022; Luttbeg & Sih, 2010; Wolf & Weissing, 2010). For example, if small individuals face greater starvation risk, they may need to be ‘bolder’ (less risk averse) and more explorative to find resources faster, even if these behaviours increase predation risk (Biro & Stamps, 2010; Sih et al., 2015). State‐dependence means behaviour is likely to be integrated within broader life‐history strategies (Dammhahn et al., 2018; Mathot & Frankenhuis, 2018; Réale, Garant, et al., 2010) and differ systematically between sexes (Patrick & Weimerskirch, 2014). For example, in some systems, males on average need to adopt riskier behaviour than females in order to obtain mating opportunities (Harris et al., 2010; Nathan et al., 2008). Conversely, female behaviour may be selected to reduce costs from male harassment (Clutton‐Brock & Parker, 1995).

Although evolutionarily motivated studies dominate the literature on animal personality (Briffa & Weiss, 2010), this phenomenon is increasingly recognised as having wider implications and applications. For example, Prentice et al. (2022) argues how the integration of personality traits with stress physiology means artificial selection on behavioural biomarkers could be used to improve welfare in fish aquaculture (see also e.g. Castanheira et al., 2013; Ibarra‐Zatarain et al., 2016). Ecotoxicology is another applied field in which the potential importance of among‐individual differences in behaviour has been recently highlighted (Bertram et al., 2022). For example, great tits (*Parus major*) with high levels of lead in their blood and high levels of multiple metals in their feathers, showed lower explorative behaviours on average (Grunst et al., 2019), while insecticide exposure lowered behavioural repeatabilities in spiders (Royauté et al., 2015), reducing the relative importance of among‐individual differences. Furthermore, Polverino et al. (2021) found long‐term fluoxetine exposure in guppies (*Poecilia reticulata*) erodes variation in activity levels between individuals. Ubiquitous contamination may, therefore, impair behaviour and future adaptive potential of phenotypic variation to anthropogenic‐induced alterations within both terrestrial and aquatic landscapes.

In decapod crustaceans, such as the species we investigate here, several applications of personality variation have been suggested. First, just as in fishes, personality traits may be relevant to welfare outcomes in captivity, which are under increasing scrutiny following recognition of sentience (Birch et al., 2021; Gherardi, 2009). Second, decapod behavioural change following sublethal exposure to environmental pollutants could contribute to bioassays relevant for monitoring ecosystem health and susceptibility of benthic and/or sediment dwelling invertebrates to pollutants (Razekenari et al., 2023). Third, since decapods demonstrate trait‐biased dispersal, with bolder individuals outcompeting conspecifics, variation in boldness and activity may link to invasive success (Galib et al., 2022; Malmqvist, 2002). Furthermore, many billions of decapod crustaceans are harvested from wild fisheries and raised in aquaculture systems for human consumption annually (Elwood, 2012). Personality differences have been shown to predict trappability in decapods (Biro & Sampson, 2015; Moland et al., 2019) just as in vertebrates (Garamszegi et al., 2009; Vanden Broecke et al., 2021), and this differential trappability may exert selection pressures on behaviour in wild populations. Finally, behavioural differences are also expected to be integrated with life‐history traits important for production in aquaculture, where, for example, traits associated with foraging can affect growth rates (Bardera et al., 2019; Daly et al., 2021).

Here we investigate the presence and structure of among‐individual variation in the red cherry shrimp *Neocaridina heteropoda* (syn. *N. davidi*), a small (<30 mm) caridean species. This is a popular ornamental species that, being easy to maintain and breed under laboratory conditions, is an emerging model for pharmaceutical and ecotoxicological research, with relevance to ecosystem stability (Bardera et al., 2019; Horvath et al., 2013; Hu et al., 2019; Pantaleao et al., 2017; Razekenari et al., 2023; Sung et al., 2011; Weber & Traunspurger, 2016). A short generation time and fast development also make it amenable to genetic studies and potentially a convenient model system for decapod aquaculture (Bondad‐Reantaso et al., 2012; Hauton, 2012). Cherry shrimp are of commercial aquaculture importance themselves as an ornamental species (Heerbrandt & Lin, 2006). Unfortunately, release by aquarists combined with a wide tolerance of water and temperature parameters means they have become invasive outside their native range (Pantaleao et al., 2017; Klotz et al., 2013; Weber & Traunspurger, 2016). Despite this, basic biological information on this species is scarce (but see e.g. Pantaleao et al., 2017; Razekenari et al., 2023). In particular there is a lack of baseline behavioural data that may, for example, impede use of ‘behavioural endpoints’ in ecotoxicology (Ågerstrand et al., 2020; Faimali et al., 2017; Melvin & Wilson, 2013). Very little is currently known about the amount or structure of behavioural variation among‐individuals, nor is it known whether state‐dependent behavioural variation, if present, is linked to intrinsic variables such as size or sex.

We focus specifically on ‘shy–bold’ variation (Wilson et al., 1993), an aspect of personality that describes differences in behavioural response to (perceived) risk (Toms et al., 2010). We use two simple testing paradigms, Open Field Trials (OFT) and Food and Shelter trials (FST), coupled with multivariate behavioural phenotyping and a repeated measures design. Our specific aims are to (i) test for repeatable among‐individual differences of the behavioural measures of boldness across the OFT and FST, (ii) determine whether the structure of multivariate behavioural variation observed is consistent with expectations given an underlying shy–bold among‐individual axis (i.e. ‘bold’ individuals explore more actively the arena, show less thigmotaxis and stress response and are more willing to feed than ‘shy’ individuals) and (iii) determine whether size and/or sex explain behavioural variation among‐individuals.

## 2 METHODS

### 2.1 Study animals and husbandry

All shrimp used were from a captive‐bred colony sourced from the aquarium pet trade in February 2022 and subsequently maintained in the Freshwater Laboratory of the Animal Facility in Penryn, Cornwall. The founding colony consisted of 200 red morph adult shrimp, with unknown sex ratio. On arrival in the laboratory, adult shrimp were housed in large breeding stock tanks (28 cm × 19.5 cm × 18.5 cm) for several months to establish the breeding colony. Behavioural data for this study were collected during October and November 2022 under the local ethical approval (University of Exeter approval ID 517031). Forty‐eight individuals were taken from stock tanks and moved to individual housing containers (22 cm × 8.5 cm × 15 cm) connected to a shared recirculating water supply. Each individual tank contained a short piece of black plastic (3.5 cm × 3.5 cm) and a plastic plant to provide refuge. Shrimp moved to these containers were sampled haphazardly from stock, but with the condition that they needed to have a body length of at least 6 mm. These were presumed to be adult females and males since individuals with a total length of >7 mm can be sexed (De Silva, 1988; Pantaleao et al., 2017; Schoolmann & Arndt, 2018) and to ensure the effective tracking of behaviour in the experimental setup (described below). Water temperature was maintained at 25°C and shrimps were fed every 2 days on commercial ORGANIX granulate shrimp pellets. A constant light: dark cycle was enforced (lighting hours 07:00–19:00).

### 2.2 Overview of experimental design

To test for and characterise among‐individual variation in *N. heteropoda* we aimed to subject each of the 48 individuals to three repeats of two separate behavioural assays: an Open field trial (OFT) and a Food and shelter trial (FST). The order of assays was held constant, with all individuals completing 3 × OFT followed by 3 × FST over a five‐week period, with a minimum of 48 h between any two successive trials. This design would have yielded 288 trials (48 individuals × 3 repeats × 2 assays). However, some mortalities occurred during the investigation period. In some cases, we opportunistically replaced mortalities with new stock shrimp such that our final data set analysed actually comprised 273 trials on 53 shrimp, with a mean of 2.5 OFT and 2.6 FST per individual. In our final sample, 43 individuals were tested in both assays (41 of which had at least 2 repeats for each assay), 5 only in the OFT and 5 only in the FST. We note that a small number of assays (5 OFT and 1 FST) took place but was discarded later for technical videotracking issues. Stress responses (e.g. to handling or changes in conditions due to the behavioural assays) can trigger moulting in crustaceans, which is known to affect behaviour (see Bacqué‐Cazenave et al., 2019). Before each trial, we recorded the presence of an exuvia from moulting in the housing tank; 23 shrimp out of 273 moulted over the experimental period. However, because this was recorded only on the day of the assay, exuviae could be remains from moulting in previous days, and we do not know how long the possible behavioural changes may persist after moult.

Trials were run between 09:00 and 13:00 h with individuals tested in a random order. On any given day, all trials conducted in the laboratory were of a single assay type, with duplicate experimental arena tanks allowing two shrimp to be tested simultaneously. These experimental tanks (30 cm × 20.5 cm × 21 cm) were filled to 5 cm^3^ with water from the sump of the recirculating water system to which all individual housing units were connected. The water was changed every 6 trials to reduce any influence of conspecific cues that might be produced. Each tank was filmed from above using a Sunkwang C160 video camera mounted with a 5–50 mm manual focus lens, and the tracking software Viewer II (BiObserve) used to measure behaviours putatively linked to shy–bold variation (described below for each assay). The experimental tanks were surrounded by cardboard screens to exclude external visual stimuli that might otherwise impact behaviour. Individuals were randomly assigned before each trial to one of the two arenas to minimise slight technical differences in setup, such as lighting and camera angle, that may affect the tracking, or possible differences, for example in outside disturbance or other conditions that may affect the behaviour of the shrimp itself.

At the end of each trial, size and sex data were recorded and the shrimp was then returned to individual housing. For size we measured both length (mean: 15.77 mm, SD: 2.28; from the tip of the rostrum to the posterior end of the telson using digital callipers to the nearest 0.01 mm) and mass (mean: 0.07 g, SD: 0.03; using a digital balance after dabbing the animal with a tissue to remove water droplets to the nearest 0.01 g). However, length and mass measures were highly correlated across observations (*r* = .717, *t*_268_ = 16.843, *p* < .001), while mass was slightly more repeatable at the individual level (R_mass_ = 0.768 vs. R_length_ = 0.581). Assuming size was (approximately) constant for individuals over study period then this suggests mass has a lower measurement error. We, therefore, used mass as our measure of size in all analyses. Sex was estimated from external morphology after each trial, and shrimp were scored as a male, female or of unknown sex. On average, females are larger and more opaque than males and have more rounded bellies and body plates (Vazquez et al., 2017). Females can sometimes be seen carrying eggs in their swimmerets and may show a distinctive ‘saddle’ marking (Serezli et al., 2017). Given uncertainty in sex determination, we elected to score it after each trial blind to any previous assessments of the same individual. After all observations were complete, we assigned a single sex determination of a male or female if ≥5/6 trial‐specific assessments were in agreement. We assigned sex as ‘unknown’ if this criterion was not met. Following this criterion, our final sample consisted of 17 males, 14 females and 22 shrimp of unknown sex.

### 2.3 Open field trials (OFT)

**FIGURE 1:**
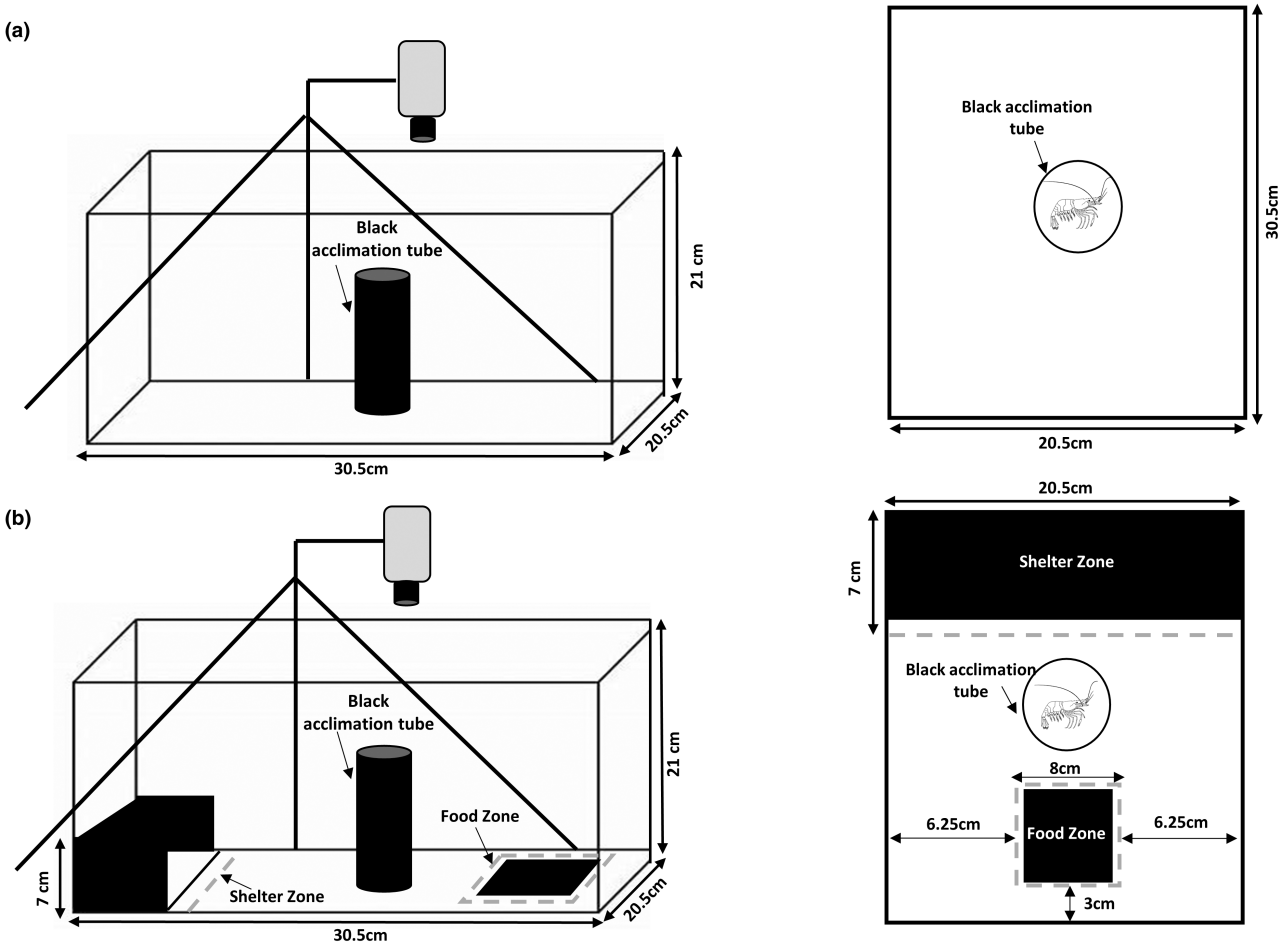
The tank setups used for (a) Open field trials (OFT) and (b) Food and shelter trials (FST) showing a side view on the left and an overhead view on the right. The starting set up for both assays has the shrimp to be tested placed inside the black acclimation tube.

The OFT is a generic and simple assay widely used across taxa to measure shy–bold variation related aspects of personality (e.g. exploration in a novel environment, anxiety‐like behaviour, stress coping style) (Carter et al., 2013; Champagne et al., 2010; Dingemanse et al., 2002). In this assay, a shrimp was placed within a tube positioned in the centre of the tank (Figure 1a) and allowed to acclimate for 120 s. The tube was then lifted out and movement tracked for a subsequent 240 s using Viewer. We extracted four behavioural traits from tracking data: *Track Length*, *Area Covered*, *Wall Distance* and *Freezings* (see Table 1 for definitions). Based on OFT behaviour in other taxa, our prediction is that bolder individuals will tend to show higher values for the first three traits (i.e. more active exploration of the arena and less thigmotaxis) but lower values for freezing (Aparicio‐Simón et al., 2010; Perrot‐Minnot et al., 2017). The latter prediction stems from the fact that freezing behaviour under perceived risk is a common component of behavioural stress response (e.g. the ‘flight‐fight‐freeze’ response) (Houslay et al., 2022). These behaviours are widely used in studies of shy–bold variation based on similar assays applied in fishes (Boulton et al., 2014; Polverino et al., 2016; Toms et al., 2010). We consider this an appropriate starting point, but fully acknowledge that a priori predictions are naïve with respect to decapod biology in general (and *N. heteropoda* specifically). Summary statistics for the original variables measured in the OFT assay are presented in Table S1.

**TABLE 1:** Behavioural traits recorded in Open field trial (OFT) and Food and shelter trials (FST).

Assay	Trait	Definition of measured behaviour (before transformation)
OFT	*Track Length*	Total distance travelled (cm)
	*Area Covered*	Proportion of total arena (%)
	*Wall Distance*	Average distance away from tank walls (cm)
	*‐(Freezings)*	Number of ‘freezes’ defined as speed dropping <4 cm s^−1^ for ≥25 s
FST	*Track Length*	Distance travelled while outside shelter and food zones (cm)
	*Time in Open*	Time spent outside the shelter zone (s)
	*‐(Food Latency)*	Time to first entry of the food zone (s)
	*‐(Freezings)*	Number of ‘freezes’ (defined above) outside shelter and food zones

### 2.4 Food and shelter trial (FST)

The FST used a modified arena into which we added (i) a shelter made of black plastic (18 cm × 5 cm × 5.5 cm), positioned at one end of the tank and (ii) a food zone, comprising a black plastic square (5 cm × 5 cm) with food glued to it at the other end (Figure 1b). The food was a mixture of Repashy Solient Green premix powder and ORGANIX shrimp granulate pellets (approximately 10) glued to the black plastic square using aquarium safe glue (see Figure 1b) to prevent floating, but still accessible to the shrimp. The food zone was re‐used between trials since the food consumption was minimal compared to the amount available, but changed at the end of each day. Shrimp were introduced via the black acclimation tube at the centre of the tank for 120 s, then tracked for 240 s after being released. Four traits were measured: *Track Length*, *Time in Open*, *Food Latency* and *Freezings*. Predictions for *Track Length* and *Freezings* are as described above but note that movement of shrimp could not be tracked once they were within the shelter and food zones, so these traits are recorded only for the portion of the observation period that they are outside these zones. We predict that bolder individuals will spend more time in the open (i.e. outside the shelter zone) and have a short latency to enter the food zone (used as a willingness to feed). Summary statistics for the original variables measured in the FST assay are presented in Table S1.

### 2.5 Statistical analyses

We used mixed effect models fitted with ASReml‐R implemented in R version 4.1.1 (R Core Team, 2023). We applied log‐transformations (OFT *Wall Distance*; FST *Food Latency*) and square root transformations (OFT *Track Length*, *Freezings*; FST *Track Length*, *Time in Open* and *Freezings*) to improve Gaussian assumptions, before scaling to standard deviation units which facilitates multivariate modelling. Finally, we also multiplied the transformed and scaled data for *Freezings* (both assays) and *Food Latency* (FST) by −1. This sign reversal was to simplify biological interpretation of results by making high values correspond to a priori expectation of ‘bolder’ behaviour in all cases. Following these transformations, model residuals were (approximately) Gaussian with the exception of *‐(Food Latency)*, which showed major departures from residual normality that could not be resolved. Strictly Gaussian residuals are assumed in generating P values for inference, but, as shown in (Schielzeth et al., 2020), linear models have proven very robust to this, and, therefore, deviations from Gaussian residuals should not cause bias in the parameter estimates presented.

### 2.6 Among‐individual variance in behavioural traits

We tested for among‐individual variation in each of the OFT and FST traits using a series of univariate linear mixed models. For each trait, we fitted a model with fixed effects of: order (from 1 to 6 reflecting the order of individuals tested between experimental water changes), trial repeat number for the individual (from 1 to 3), time of day (in minutes after midnight) and experimental arena used (tank A vs. B). The FST traits of *Track Length* and *‐(Freezings)* are analogous to OFT traits but were only recorded for the portion of the observation period while shrimp were trackable outside the food and shelter zones. Since both traits were square root transformed for analysis, we included the square root of time spent in the trackable part of the arena as an additional fixed effect in the model of these traits. All these fixed effects were included simply to control for potential nuisance variables unrelated to our hypotheses. Each model also contained a random effect of individual identity (ID), allowing us to estimate among‐individual variance *V*_I_. For each trait, we then estimated repeatability (R) conditional on fixed effects as the proportion of phenotypic variance (*V*_P_) explained by individual differences. Thus R = *V*_P_/(*V*_I_ + *V*_R_), where *V*_R_ is the residual (within‐individual) variance. For each trait we compared our model to a reduced version of the same model without the random effect of individual identity by likelihood ratio test (LRT) to assess the significance of *V*_I_. For testing a single variance component, we assumed twice the difference in log‐likelihoods is distributed at a 50:50 mix of *χ* ^2^ on 0 and 1 DF following (Visscher, 2006).

### 2.7 Among‐individual covariance in behavioural traits

Next, we fitted a multivariate mixed model to estimate the among‐individual behavioural covariance matrix (**ID**) for the full set of 8 traits. Fixed and random effects on each trait were as described above for the univariate models. **ID** contains estimates of *V*_I_ for each trait on the diagonal, with off‐diagonal elements corresponding to COV_I_, the among‐individual covariance for each pair of traits. Residual within‐individual (co)variance was partitioned to the corresponding matrix **R**. However, residual covariance (COV_R_) is only identifiable between trait pairs observed simultaneously (i.e. in the same trial), so was fixed to zero between OFT and FST traits. To test the presence of among‐trait covariance in **ID**, we compared the full model to one in which all COV_I_ were fixed to zero by LRT assuming twice the difference in model log‐likelihoods distributed as $\chi_{28}^{2}$.

Having estimated **ID**, we then wanted to assess whether it was qualitatively consistent with a dominant underlying axis of shy–bold variation as we predicted. To do this we (i) standardised among‐individual covariance terms to the more intuitive correlation scale (where, for any pair of traits x,y the among‐individual correlation *r*_I(x,y)_ = COV_I(x,y)_ / √(*V*_Ix_ × *V*_Iy_)); and (ii) subjected our estimated matrix to eigen decomposition (principal component analysis). Since all traits were transformed such that high values indicated bolder behaviour, we predict correlations should be uniformly positive. We also predict that the leading eigen vector of **ID** (subsequently referred to as **id**_**max**_) will explain a large proportion of among‐individual variance and have same‐sign loadings on all traits. We used a parametric bootstrap approach, following (Boulton et al., 2015) with a bootstrap sample size of 1000, to generate approximate 95% CI on the eigen values of **ID** and trait loadings on **id**_**max**_.

### 2.8 Testing whether sex and size contribute to among‐individual variation

To assess the extent of the contribution of sex and/or size to the among‐individual variation in behaviour, we refitted all models described above but with additional fixed effects of sex (a 3 level factor: male, female, unknown), size (mass as a covariate) and their interaction sex:size. Using univariate models and conditional F‐tests, we tested the significance of these effects on each trait. We compared estimates of *V*_I_ and R from these expanded models to those obtained above. We then refitted our multivariate model with the additional fixed effects and estimated **ID**_**sex:mass**_, the among‐individual (co)variance matrix conditional on sex and mass. We scaled covariances to correlations and subjected **ID**_**sex:mass**_ to eigen decomposition as described above, allowing us to compare its structure to **ID**. We also checked for any clustering of personality visually by sex and/or size in a multivariate personality space, by predicting and plotting individual scores on **id**_**max**_ for each shrimp.

## 3 RESULTS

### 3.1 Among‐individual behavioural variation

**FIGURE 2:**
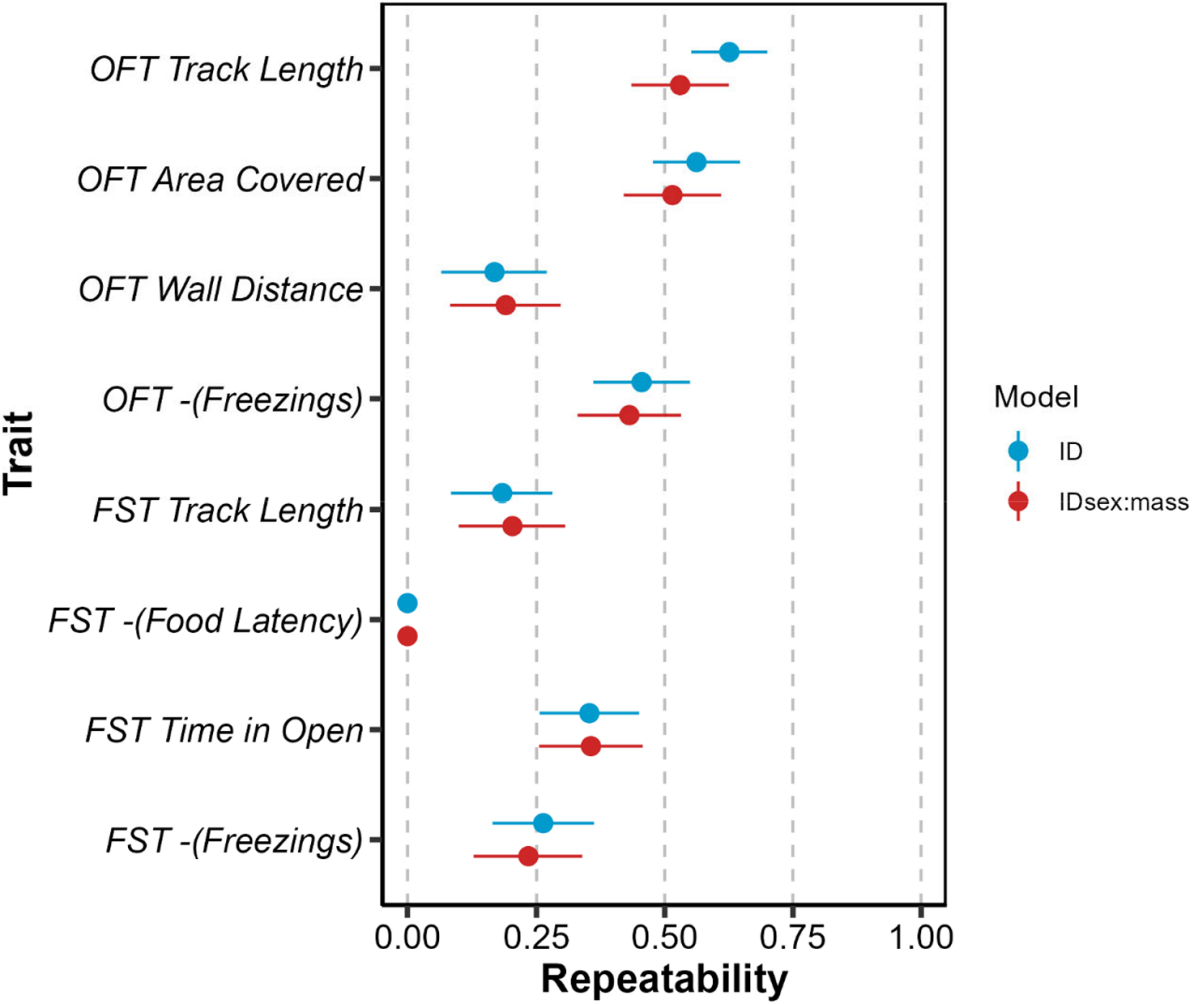
Repeatabilities for OFT and FST traits estimated from initial univariate models (blue points) and refitted models that condition on additional fixed effects of size and sex (red points). Error bars depict estimates ±1SE.

Our shrimp population shows significant among‐individual differences in all behavioural traits measured in OFT and FST assays bar *‐(Food Latency)* (Table 2). Thus, behaviours are repeatable with a median of R = 0.353 (range 0.168–0.626) conditional on fixed effects (Figure 2, blue points). Fixed effect estimates from these models are not relevant to biological hypotheses but are presented in the Supplementary Material (Table S2). Of these, we note that replicate (i.e. number of repeat) affected OFT *Area Covered*, OFT *Wall Distance* and FST *Freezings*, possibly indicating an habituation effect over time.

**TABLE 2:** Estimated variance components and repeatabilities (R) from univariate mixed effect models of OFT and FST traits.

Assay	Trait	*V*_I_	*V*_R_	R	$\chi_{0,1}^{2}$	*p*
OFT	*Track Length*	0.616 (0.158)	0.368 (0.058)	0.626 (0.074)	48.884	<.001
	*Area Covered*	0.529 (0.146)	0.413 (0.065)	0.562 (0.085)	33.988	<.001
	*Wall Distance*	0.153 (0.100)	0.759 (0.118)	0.168 (0.102)	3.040	.041
	*‐(Freezings)*	0.456 (0.141)	0.546 (0.086)	0.455 (0.094)	22.633	<.001
FST	*Track Length*	0.102 (0.06)	0.458 (0.069)	0.183 (0.098)	4.032	.022
	*Time in Open*	0.365 (0.13)	0.668 (0.102)	0.353 (0.097)	14.207	<.001
	*‐(Food Latency)*	0.000^a^	0.995 (0.122)	0.000^a^	0.000	.500
	*‐(Freezings)*	0.109 (0.048)	0.306 (0.046)	0.263 (0.099)	8.056	.002

*Note*: *V*_I_ and *V*_R_ denote among‐individual and residual variances, respectively, and standard errors are shown in parentheses. Also shown are *χ* ^2^ and *p* derived from LRT to test the significance of *V*_I_ for each trait.

^a^ For this trait the estimate of *V*_I_ was bound to zero and no SE is estimable.

### 3.2 Among‐individual (co) variation

**FIGURE 3:**
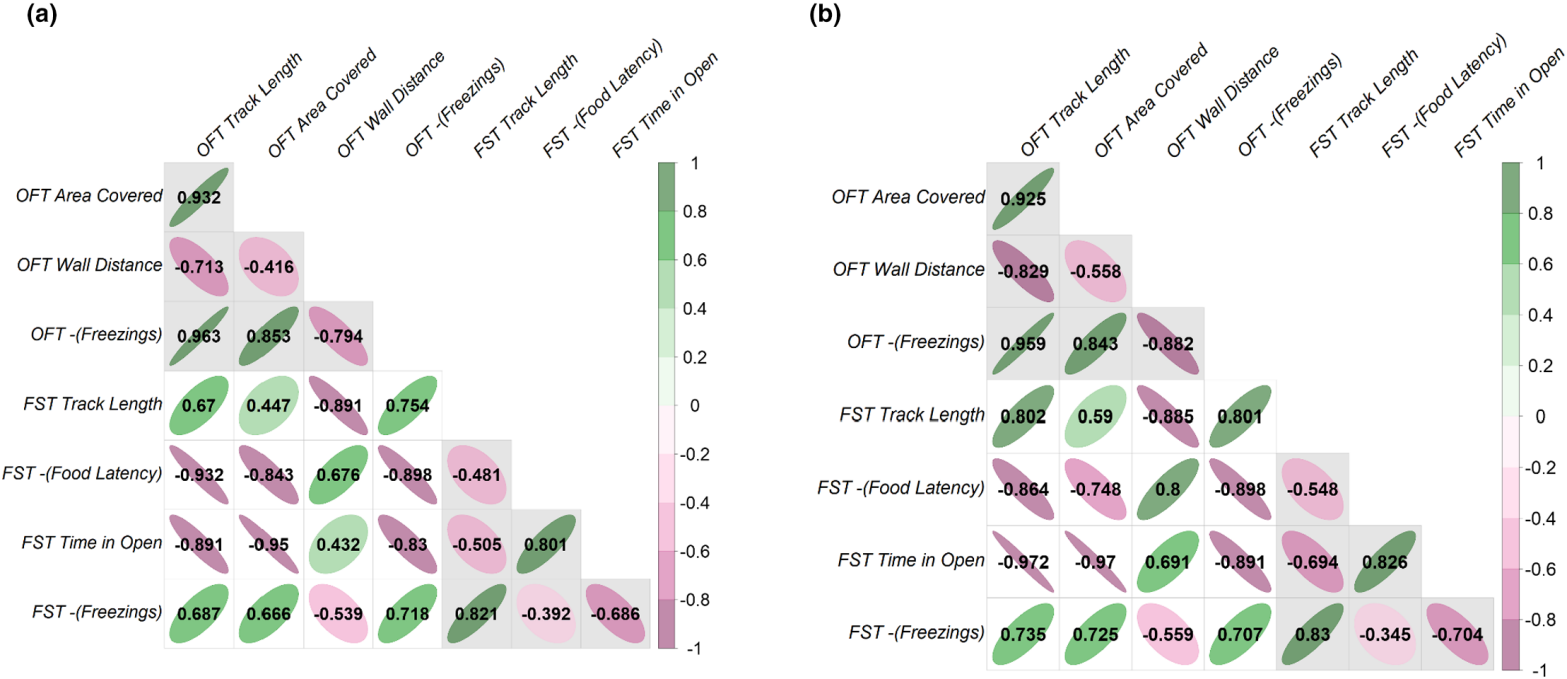
Among‐individual correlation structure between traits as determined from multivariate model estimates of (a) **ID** the variance–covariance matrix and (b) **ID**_**sex:mass**_, the corresponding matrix conditional on sex and size. Ellipse shape and colour denote the strength and sign of each correlation. Light grey background shading indicates sets of correlations among traits measured within each assay type (FST, OFT), while across‐assay correlations have white backgrounds.

**FIGURE 4:**
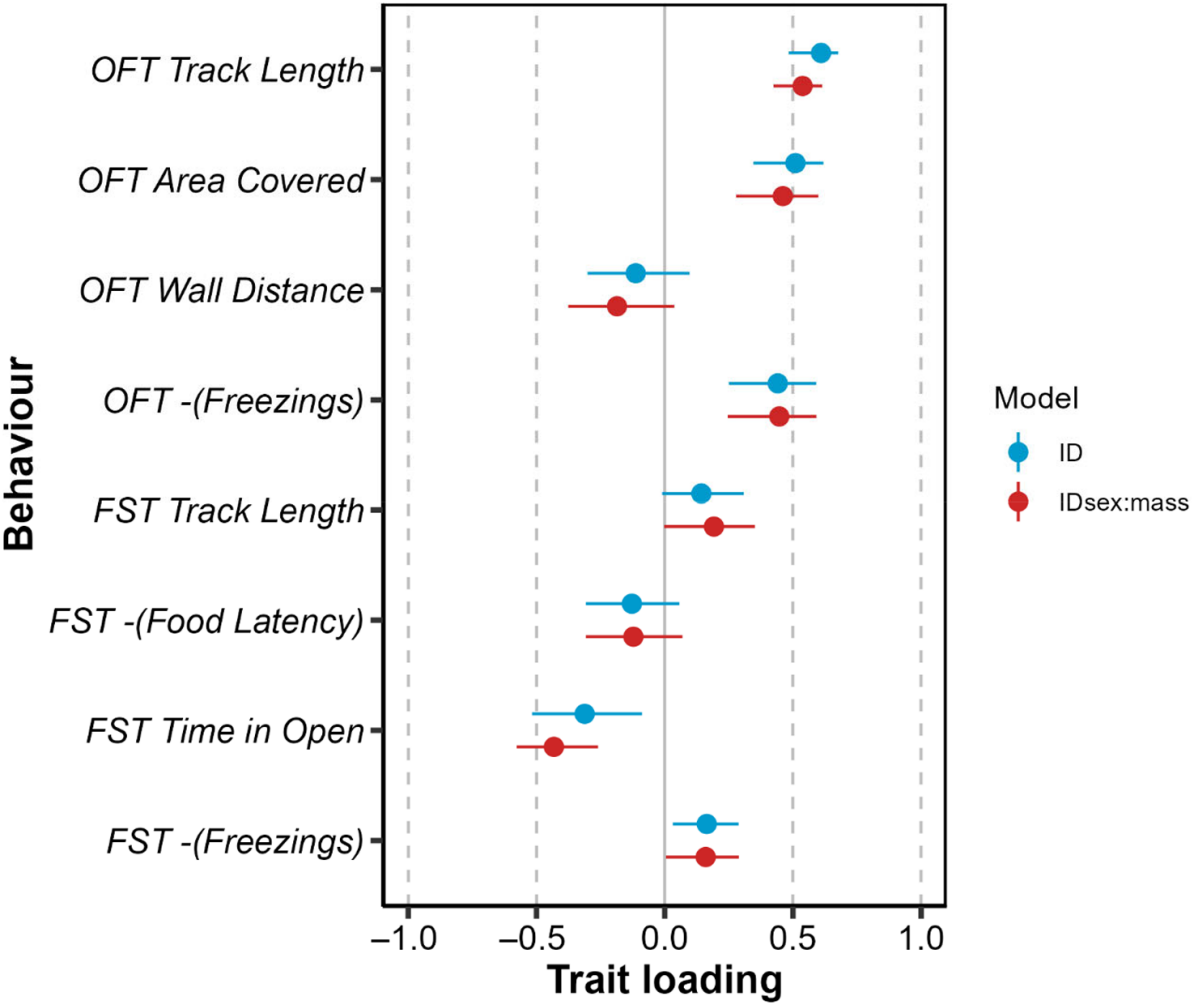
Trait loadings on the leading vectors of **ID** (blue points) and **ID**_**sex:mass**_ (red points). Error bars denote 95% confidence intervals obtained using a parametric bootstrap (*n* = 1000) and loadings can be considered nominally significant if CI do not cross zero (solid vertical line).

The multivariate model provides statistical support for individual‐level covariance among the traits tested (LRT comparison of full model to one in which all COV_I_ terms fixed to zero; $\chi_{28}^{2}$ = 104.87, *p* < .001). Pairwise correlations between traits in **ID** are generally strong (Figure 3a) with a median absolute magnitude of 0.736 (range −0.95 to 0.963). Furthermore, correlations among traits within‐ and across‐ assays are of similar magnitude. However, contrary to our prediction of a simple shy–bold axis of variation, the among‐individual correlations between (transformed) trait pairs are not uniformly positive (Figure 3a). This result is also reflected in the eigen decomposition where **id**_**max**_ captures 61% (95% CI, 46.82%–72.23%) of the among‐individual (co)variance, consistent with a strong axis of personality underpinning the observed behaviours, but loads antagonistically on some traits (Figure 4, blue points). These loadings show that individuals appearing bolder than average as measured by most traits (e.g. OFT *Track Length*, OFT *‐(Freezings)*), tend to appear less bold than average as measured by OFT *Wall Distance*, FST *‐(Food Latency)* and FST *Time in Open*. Of these traits, only FST *Time in Open* clearly loads significantly on **id**_**max**_ (based on 95% CI not overlapping zero), but this is particularly notable as it indicates shrimp we might view as bolder based on most other criteria actually spend more time than average in the shelter during FST. We present the full **ID** variance–covariance matrix from which the correlations are derived in Table S3. We note that as V_i_ was small and non‐significant for FST ‐(*Food Latency*), we are cautious when interpreting correlations with this trait.

### 3.3 Effects of sex and mass

**FIGURE 5:**
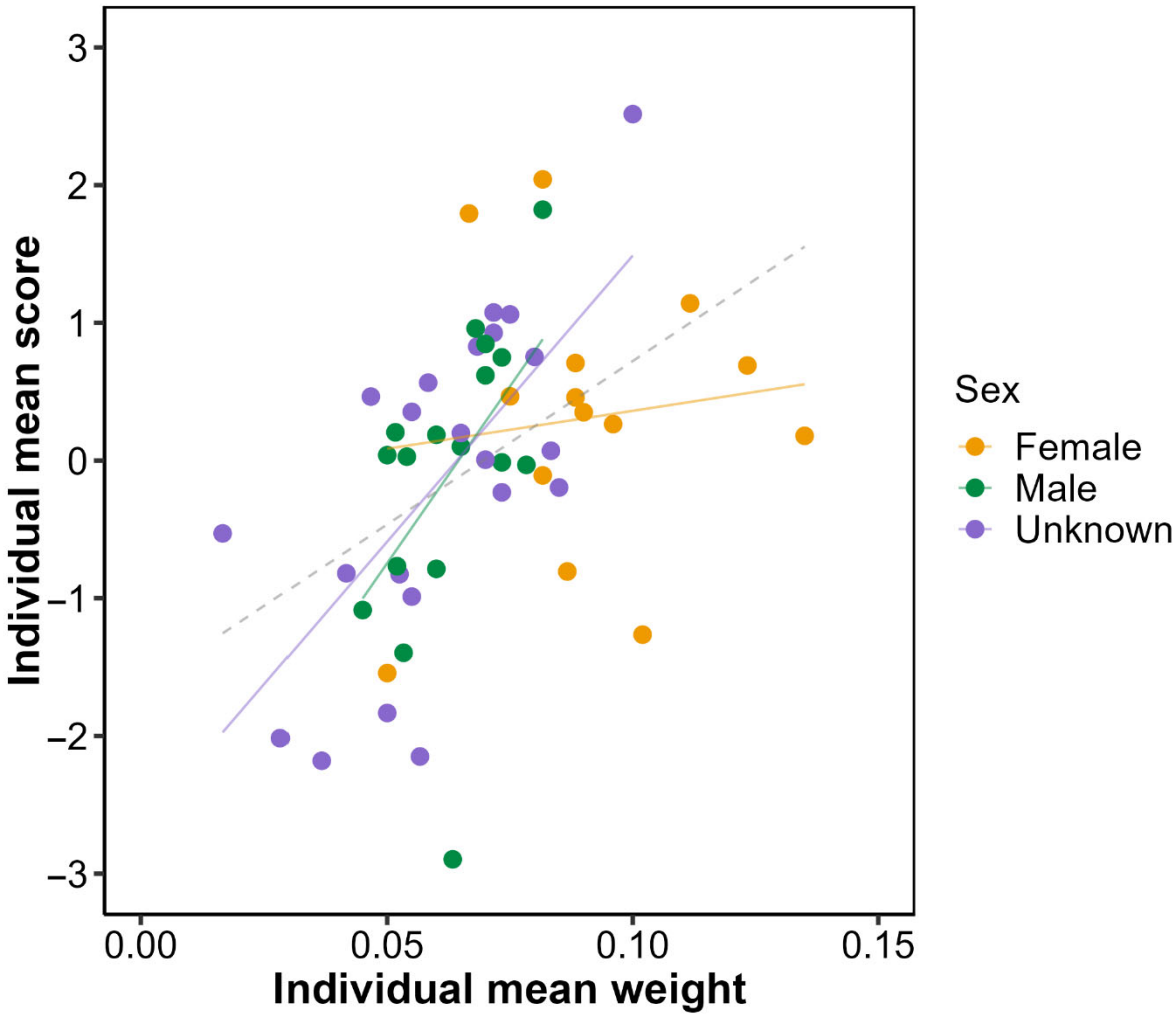
A graphical illustration of the relationship between multivariate personality, mass, and sex. Each point represents an individual's predicted behavioural score **id**_**max**_ plotted against mean weight, with colours denoting assigned sex (female, male, unknown). Behavioural scores are calculated for each individual as **id**_**max**_ **.i** ^T^ where **i** is a column vector containing the best linear unbiased predictions (BLUPs) of individual deviations from each trait mean. Also shown for illustrative purpose are overall (grey dashed) and sex‐specific regressions of behavioural score on mean weight. Behavioural scores are (uncertain) model predictions and no statistical inference is intended.

We find limited support for the hypothesis that among‐individual behavioural differences are explained by sex and/or size (mass). Refitted univariate models provide no statistical support for differences in behavioural means between assigned sexes (Table 3), nor did we detect any significant sex:size interactions. However, we did detect significant main effects of size (mass) on OFT *Track Length* and OFT *Area Covered*. Both coefficients are positive meaning heavier individuals on average cover longer distances and a larger area in OFT trials. Estimates of *V*_I_ and R from these refitted univariate models were very similar to those estimated unconditional on the extra fixed effects (Figure 2), providing further confirmation that neither sex nor size affect considerable among‐individual behavioural variation in single traits. This same result also holds for the multivariate phenotype in that the correlation structure in **ID**_**sex:mass**_ is very similar to that in **ID** (Figure 3). Moreover, the first vector of **ID**_**sex:mass**_ captures 65% (95% CI 47.96%–73.26%) of multivariate variance and has trait loadings almost identical to those of **id**_**max**_ (Figure 4). Finally, plotting individual behavioural scores on **id**_**max**_ confirms the absence of clustering by sex (Figure 5). While a net positive association between mean weight and **id**_**max**_ can be seen, it is also clear that size accounts for only a small fraction of the variation present. We present the full table of fixed effects estimates in Table S4 and the full **ID**_**sex:mass**_ variance–covariance matrix from which the correlations are derived in Table S5.

**TABLE 3:** Estimated effects of sex and size on OFT and FST behavioural traits.

Assay	Trait	Effect	Level	Coefficient (SE)	*F*	df	*p*
OFT	*Track Length*	Sex	Female	1.523 (0.862)	0.271	2,44.7	.76
			Male	1.246 (0.774)			
		Mass		12.38 (6.131)	7.049	1111.6	.009
		Sex:mass	Female	−3.00 (10.14)	0.058	2115.8	.944
			Male	0.792(11.61)			
	*Area Covered*	Sex	Female	−1.913 (0.876)	0.072	2,45.8	.931
			Male	−1.870 (0.788)			
		Mass		18.40 (6.232)	4.608	1110.4	.034
		Sex:mass	Female	−17.09 (10.31)	2.073	2115	.130
			Male	−19.43 (11.84)			
	*Wall Distance*	Sex	Female	−0.327 (0.885)	0.251	2,44.8	.779
			Male	−0.907 (0.853)			
		Mass		1.985 (6.303)	0.044	1,74.9	.834
		Sex:mass	Female	−9.506 (10.49)	0.437	2,84.3	.648
			Male	−0.510 (13.02)			
	*‐(Freezings)*	Sex	Female	2.735 (0.939)	0.192	2,46.5	.826
			Male	2.852 (0.860)			
		Mass		12.195 (6.697)	0.764	1101.1	.384
		Sex:mass	Female	−13.75 (11.09)	1.612	2108.1	.204
			Male	−21.00 (13.00)			
FST	*Time in Open*	Sex	Female	−0.717 (0.913)	2.076	2,49.1	.136
			Male	1.306 (0.996)			
		Mass		−0.608 (5.973)	0.081	1,90.6	.776
		Sex:mass	Female	11.77 (10.40)	1.528	2,95.6	.222
			Male	−15.54 (15.26)			
	*‐(Food Latency)*	Sex	Female	−2.088 (0.775)	0.766	2127	.297
			Male	0.343 (0.937)			
		Mass		−0.939 (5.300)	0.025	1127	.874
		Sex:mass	Female	10.698 (8.915)	2.586	2127	.079
			Male	−23.32 (14.52)			
	*Track Length*	Sex	Female	−3.449 (0.666)	0.157	2,49.3	.855
			Male	−2.915 (0.756)			
		Mass		5.909 (4.441)	0.267	1,75.6	.607
		Sex:mass	Female	−7.836 (7.673)	1.465	2,85.5	.237
			Male	−18.36 (11.65)			
	*‐(Freezings)*	Sex	Female	0.187 (0.573)	0.818	2, 48.8	.447
			Male	0.028 (0.645)			
		Mass		1.587 (3.804)	0.937	1,77.5	.336
		Sex:mass	Female	2.223 (6.591)	0.203	2,87	.817
			Male	6.008 (9.93)			

*Note*: Sex was fitted as 3 level factor (female, male, unknown) with unknown treated as the reference level. Size was measured as live mass (g). Estimates are from univariate models with significance tested using conditional *F*‐tests.

## 4 DISCUSSION

Our repeated measures design provided strong statistical support for consistent among‐individual differences in behaviour across the Open field (OFT) and Food and shelter (FST) assays. As expected, multivariate analyses also yielded evidence of strong individual‐level correlations among the specific traits assayed. However, in asking whether the structure of multivariate behavioural variation was consistent with a priori expectations of an underlying axis of shy–bold personality variation, our results provided a somewhat mixed picture. Specifically, we did find a dominant leading eigen vector of **ID** that explains most (61%) of individual‐level variation in, and covariation‐among, traits. However, several of the behaviours measured load antagonistically on this axis, a pattern that does not match our a priori predictions for a simple shy–bold axis. Further analyses provided little statistical support for major contributions of size or sex effects to the personality variation described (though some size effects were found). Below we discuss each of these main results in the context of the existing literature and highlight some important caveats to our conclusions.

Behavioural repeatabilities were statistically significant in seven of the eight behavioural traits tested. This confirms the presence of personality variation in red cherry shrimp and shows it is readily detected using simple testing paradigms adapted from fish models. Estimates of R were lower in the FST assay than in the OFT assay, notably for *Track Length*. This may result in some way from the greater environmental complexity of the FST or could conceivably be due to our set up that only allowed individual tracking outside the shelter (e.g. if individual activity was more repeatable in the shelter). It would be useful to develop an assay that allows tracking of animals in the shelter, determining whether patterns of activity change systematically between being in the shelter and the open.

Although personality has yet to be widely tested in decapods, our repeatability estimates align with the conclusions of Bridger et al. (2015) who demonstrated among‐individual variation in startle response duration (a proxy of boldness) in male hermit crabs (*Pagurus bernhardus*). Repeatability in traits associated with shy–bold and/or exploratory personality variation has also been demonstrated in the European crayfish (*Astacus astacus*; Vainikka et al., 2011) and the rock pool shrimp (*Palaemon elegans*; Maskrey et al., 2018). In these latter examples, boldness was also negatively correlated with resource holding potential, suggesting a trade‐off whereby bolder individuals may be better at finding resources but less able to defend them in competition (Maskrey et al., 2018). We do not yet know if similar relationships hold in *N. heteropoda,* but investigating the functional significance of personality in relation to competition and other ecological processes (López et al., 2005) in this species would be useful. More generally, testing for associations of behavioural profiles with individual life histories would permit scrutiny of whether personality variation is maintained through adaptive processes (e.g. resource allocation trade‐offs (Dingemanse & Wolf, 2010)) and/or arises through differences in resource acquisition coupled with state‐dependent behaviour (Haave‐Audet et al., 2022).

While estimates clearly varied among traits, some repeatabilities were notably high in comparison to the wider animal personality literature. For example, estimates of R = 0.63 and 0.56 for OFT *Track Length* and *Area Covered* are high compared to a median behavioural repeatability of 0.37 reported by Bell et al. (2009). This may, at least in part, reflect the use of a short inter‐observation interval; R is known to decline as the time between observations increases (e.g. Boulton et al., 2014). However, high repeatabilities also have some implications worth noting. First, R sets an upper limit for heritability (and so potential rate of selection response) and facilitates more accurate selective breeding in aquaculture (Falconer & Mackay, 1996). Selection on behaviour has been suggested as a way to improve growth and other production traits in commercial shrimp cultivation (Bardera et al., 2019), and in this context, high repeatability of the selection target would be advantageous. In a study of Pacific white shrimp (*Litopenaeus vannamei*), Sanchez et al. (2005) found that individuals interacting more with feed had a lower latency of approach and consumed food more rapidly. In our FST assay, latency to enter the zone containing food in the FST was actually not repeatable (and so not heritable). However, if simple behavioural biomarkers of improved feeding could be identified, then selecting on these could be valuable in an industry where food waste is a major source of economic inefficiency (Cuzon et al., 2004; Sick et al., 1973). At the same time, boldness is often positively correlated with aggressiveness in animals generally (Garamszegi et al., 2013), and while agonistic behaviours appear rare in red cherry shrimp (personal observations) this is not generally true of decapods. Therefore, care must be taken that selection on behaviour to improve feeding efficiency does not exacerbate welfare and/or production costs by increasing aggression among conspecifics.

Although the implications are perhaps less clear at present, personality variation has been widely linked to the likelihood of establishment and/or invasive spreading of species following accidental or intentional introduction (Chapple et al., 2012; Rehage et al., 2016). For instance in the American signal crayfish (*Pacifastacus leniusculu*s), Daniels and Kemp (2022) found repeatable differences in shy–bold type behaviour that predicted individual motivation to disperse by passing through weirs (though not successful at doing so). Invasion processes could, therefore, be considered a ‘selective filter’ whereby only individuals with appropriate combinations of personality and other traits can invade a novel environment successfully (Chapple et al., 2022). Working on the same species of crayfish, Pintor et al. (2008), found that invasive populations were bolder and more aggressive on average than populations within their native range. However, this was only true where invasive populations were allopatric to native crayfish, and the extent to which differences were present prior to invasions, as opposed to emerging after as adaptations to a new environment, is unclear (Pintor et al., 2008). Moreover, whether population level repeatability of shy–bold type behaviours (as opposed to individual or population mean) influences invasive potential is unresolved. Low repeatability implies high plasticity‐ at least relative to ‘fixed’ individual differences‐ and there is a long‐standing hypothesis that plasticity plays an important role in adaptation to novel environments following dispersal (Baldwin, 1896). A recent study found differences in behavioural repeatability between native and invasive species of nudibranchs that is consistent with this idea (Macali et al., 2023), but more empirical studies are needed to see if low behavioural repeatabilities reliably predict risk of invasive spread.

Although we found strong support for personality in red cherry shrimp, multivariate analyses show that the structure of behavioural variation differs somewhat from our initial predictions. Our eigen decomposition of the **ID** matrix is consistent with the presence of a latent personality axis that describes the majority of among‐individual (co)variation in the measured behavioural traits (Houslay et al., 2018; White et al., 2020). This was expected: all traits analysed were chosen precisely because they are putatively measures of the same underlying shy–bold personality axis. Thus, the important result here is not the presence of correlation structure in **ID**, but the overall ‘shape’ of that structure. Specifically, because traits were scaled such that higher numbers denoted putatively bolder phenotypes, we had also predicted that covariances in **ID** would be uniformly positive, and all traits would load on the main eigenvector with concordant signs. Some, but not all, relationships were as expected. For example, individuals who travelled further than average in the OFT, also have higher track length in the FST, cover more area (OFT) and freeze less (both assays). These behavioural characteristics meet a priori expectations for the bolder end of the shy–bold continuum indicating that exploratory individuals are associated with the propensity to take greater risks (Toms et al., 2010). However, the same individuals also tend to swim closer to the tank walls in the OFT (i.e. be more thigmotaxic) and take longer to visit the food zone in the FST, which are characteristics typically associated with shy personality types. Neither of these traits load significantly on **id**_**max**_ (based on 95% CI not overlapping zero) and among‐individual variance for FST *‐(Food Latency)* was not statistically supported in the univariate model. However, FST *Time in Open* loads significantly on **id**_**max**_ with a negative sign and is also moderately repeatable. This actually means that individuals considered bolder and/or more exploratory in the OFT spend longer durations than average in the shelter during FST, a result that is counterintuitive. Very speculatively, it is possible that the shelter provided may have been perceived as a risky environment (rather than a safe one as intended). This could arise if, for instance, our shelter mimicked the type of structure used by drift‐feeding and opportunistic foraging predatory fish (Nunn et al., 2011; Willis et al., 2019). With this considered, it would be interesting to investigate whether a refuge with greater structural complexity and/or smaller open spaces may be preferred (as demonstrated in mud crabs *Scylla serrata*; Mirera & Moksnes, 2013).

We find limited evidence for sex and size effects on behaviour and conclude that these aspects of state do not make a major contribution to personality (co)variation in our population. The absence of sex effects is perhaps somewhat surprising given the extensive evidence of behavioural sexual dimorphism in decapods. For example, male rock pool prawns (*Palaemon elegans*) are bolder and more active than females (Chapman et al., 2013), while aggression is sexually dimorphic in white shrimp (*Penaeus vannamei*; Chow & Sandifer, 1991), rock shrimp (*Rhynchocinetes typus*; Dennenmoser & Thiel, 2007) and American lobsters (*Homarus americanus*; Karavanich & Atema, 1998). In these species, males tend to be more aggressive and able to monopolise food for longer durations relative to females. In finding an absence of sexual dimorphism here we acknowledge that uncertainty in sexing the shrimp reduces statistical power. Of the 53 shrimp tested, we ultimately classified 22 as being of unknown sex. These were, on average, smaller individuals than those assigned to male or female categories and likely to be younger (and potentially sexually immature). We, therefore, cannot exclude the possibility that our study partially conflates sex, age, and maturation status in ways that mask any dimorphism and thus interpret our results with caution. To check whether our findings may have been affected by the high number of unknown individuals, we re‐run the univariate models with sex, mass, and their interaction, removing the individuals of unknown sex (noting that the sample size decreases considerably). For all the traits, sex and sex:mass interactions were not significant (results not shown). Nevertheless, we also note that several other decapod studies have reported an absence of sex effects on shy–bold type traits. For example, Brodin and Drotz (2014) found no difference in mean boldness or activity between male and female Chinese mitten crabs *Eriocheir sinensis*, while sex did not predict startle response duration in hermit crabs *Pagurus bernhardus* (Briffa et al., 2008).

We did find some evidence for size‐dependent behaviour. Statistical support was limited to two traits in the OFT, with larger (heavier) individuals travelling slightly further and covering more area on average. These trait‐specific effects drive a trend towards larger individuals having higher behaviour scores on **id**_**max**_. This could potentially be explained by links between behavioural type and life‐history strategy as proposed under heuristic frameworks such as the ‘pace‐of‐life syndrome’ (Biro & Stamps, 2008; Réale, Garant, et al., 2010). For instance, high metabolic rate may be associated with bolder behaviour, increased resource acquisition and faster growth leading to increased size (Careau et al., 2008), albeit at the likely cost of higher mortality risk (e.g. from predation; Wolf et al., 2007). At present we lack individual‐level data on life histories to test these hypothesised relationships. We also lack sufficient ecological data to explore (arguably) simpler explanations for size‐dependence. For instance, Toscano et al. (2014) argued size‐dependent behaviour of mud crabs (*Panopeus herbstii*) was linked to size‐dependent predation risk. Small crabs used refuges more than large crabs and also increased use in the presence of predators. Other mechanisms proposed for generating size‐behaviour relationships in decapods are related to mating traits. For instance, male–male competition can drive size‐dependent mating tactics (Correa & Thiel, 2003) with larger males engaging more in mate guarding (Knolton, 1980) and contest behaviour (Jivoff & Hines, 1998; Wilber, 1989) while smaller rivals adopt exploratory mate searching tactics (Correa & Thiel, 2003). Although we cannot yet rule out similar processes in cherry shrimp, it is notable we found no sex by size interactions on the behaviours assayed here (i.e. both larger males and females have higher *Track Length* and *Area Covered* in the OFT).

In conclusion, this is the first study to our knowledge that describes patterns of among‐individual variation in putative measures of shy–bold variation in the cherry shrimp, *N. heteropoda*. Using simple assays widely applied to small fish models, our results are consistent with the verbal model of a single major personality axis underlying among‐individual differences observed. However, the structure of this axis does not fully match a priori expectations of shy–bold personality. This could be because our initial assumptions of what constitutes ‘riskier’ behaviour in this species are incorrect. We also found no evidence for sexual dimorphism in behaviour and only limited support for size‐behaviour relationships. Our description of personality variation in red cherry shrimp adds to the growing picture of this phenomenon in invertebrates generally (Kralj‐Fišer & Schuett, 2014) and decapod crustaceans specifically (Gherardi et al., 2012).

While in this study we investigated the structure of behavioural (co)variation over time and two experimental contexts, it will be interesting to assess this structure's stability over ecologically relevant factors, for example the presence of predator cues (i.e. perceived predation risk) and/or anthropogenic pollutants known to impact behaviour of aquatic organisms (e.g. pharmaceuticals; Bertram et al., 2022). Such factors are widely known to impact mean behaviour, but can also alter the magnitude and stability of differences between individuals, resulting in environmental‐sensitivity of behavioural repeatabilities and the **ID** matrix (e.g. Polverino et al., 2021; Royauté et al., 2015). We have now initiated experimental work to test for these in red cherry shrimp. We therefore hope this study will set the foundations for future investigations of mechanisms and functional significance in this emerging model system, as well as provide baseline data for more applied research across ecotoxicology, aquaculture, and invasion biology.

## AUTHOR CONTRIBUTIONS

**Rosie Ann Rickward:** Conceptualization (equal); data curation (lead); formal analysis (lead); investigation (lead); methodology (lead); writing – original draft (lead); writing – review and editing (lead). **Francesca Santostefano:** Conceptualization (equal); data curation (supporting); formal analysis (supporting); investigation (supporting); methodology (supporting); supervision (supporting); validation (supporting); visualization (lead); writing – original draft (supporting); writing – review and editing (supporting). **Alastair James Wilson:** Conceptualization (equal); formal analysis (equal); investigation (supporting); methodology (supporting); project administration (lead); resources (lead); supervision (lead); validation (equal); writing – original draft (supporting); writing – review and editing (supporting).

## FUNDING INFORMATION

FS was supported by the European Union's Horizon 2020 research and innovation programme under the Marie Sklodowska‐Curie Individual Fellowship (grant agreement no. 101023262).

## CONFLICT OF INTEREST STATEMENT

The authors declare no competing interests.

### OPEN RESEARCH BADGES

This article has earned an Open Data badge for making publicly available the digitally‐shareable data necessary to reproduce the reported results. The data is available at https://doi.org/10.5061/dryad.2547d7wz2.

## Supporting information


Data S1.

## Data Availability

The dataset and associated metadata used for the analyses presented in this paper are available in the Dryad repository https://doi.org/10.5061/dryad.2547d7wz2. R Scripts used for data analysis are also available there.
