# Supplementary material for: Among‐individual behavioural variation in the ornamental red cherry shrimp, Neocaridina heteropoda
Source: Ecol Evol. 2024 Feb 22;14(2):e11049. doi: 10.1002/ece3.11049 (PMC10883255; doi:10.1002/ece3.11049)
Supplement: Supplementary file 1 — Data S1. [file ECE3-14-e11049-s001.docx]

**SUPPLEMENTARY TABLES**

**Table S1.** Summary statistics for the OFT and FST untransformed behavioural traits.

| Assay | Trait | mean | sd | median | min | max |
| --- | --- | --- | --- | --- | --- | --- |
| OFT | *Track Length* | 707.99 | 365.15 | 703.90 | 58.5 | 1752.2 |
| OFT | *Area* | 38.78 | 16.29 | 39.90 | 0.7 | 71.2 |
| OFT | *Wall Distance* | 2.71 | 1.26 | 2.40 | 0.8 | 9.1 |
| FST | *Freezings* | 10.00 | 6.34 | 10.00 | 0 | 31.0 |
| FST | *Track Length* | 147.85 | 135.56 | 104.80 | 0 | 667.3 |
| FST | *Time in Open* | 84.83 | 75.09 | 53.45 | 0 | 240.0 |
| FST | *Food Latency* | 90.72 | 103.67 | 33.10 | 0 | 240.0 |
| FST | *Freezings* | 3.05 | 3.75 | 2.00 | 0 | 19.0 |
| - | size (mm) | 15.77 | 2.28 | 16.00 | 9.00 | 21.54 |
| - | mass (g) | 0.07 | 0.03 | 0.07 | 0.01 | 0.14 |

**Table S2.** Estimated effects of experimental tank set up, order, replicate, and time of day on OFT and FST behavioural traits. Tank set up was fitted as a two-level factor (Tank A, Tank B) with Tank A being treated as the reference level. Order (1-6) reflects the order shrimp were tested between water changes. Replicate reflects the trial repeat number for the individual (from 1-3). Time of day is recorded as minutes after midnight. Estimates are derived from univariate models with significance tested using conditional F tests.

| **Assay** | **Trait** | **Effect** | **Level** | **Coefficient (SE)** | **F** | | **DF** | **P** |
| --- | --- | --- | --- | --- | --- | --- | --- | --- |
| OFT | *Track Length* | Set Up | Tank A | 1.884 (1.186) | 0.154 | | 1,94.5 | 0.695 |
|  |  |  | Tank B | 1.836 (0.122) |  | |  |  |
|  |  | Order |  | 0.012(0.038) | 0.102 | | 1, 92.6 | 0.75 |
|  |  | Replicate | | -0.100 (0.067) | 2.197 | | 1,86.5 | 0.142 |
|  |  | Time of Day | | -0.003(0.002) | 2.044 | | 1,110.8 | 0.156 |
|  | *Area Covered* | Set Up | Tank A | -2.237 (1.231) | 14.8 | | 1,96 | 0.000 |
|  |  |  | Tank B | -1.743(0.128) |  | |  |  |
|  |  | Order |  | -0.016 (0.039) | 0.155 | | 1, 93.8 | 0.695 |
|  |  | Replicate | | 0.210 (0.071) | 8.728 | | 1, 85.7 | 0.004 |
|  |  | Time of Day | | 0.002 (0.002) | 1.658 | | 1, 114.3 | 0.200 |
|  | *Wall Distance* | Set Up | Tank A | -0.975 (1.439) | 8.881 | | 1, 117.0 | 0.004 |
|  |  |  | Tank B | -1.454 (0.161) |  | |  |  |
|  |  | Order |  | 0.022 (0.050) | 0.209 | | 1, 114.1 | 0.648 |
|  |  | Replicate | | -0.233 (0.094) | 6.064 | | 1, 89.2 | 0.016 |
|  |  | Time of Day | | 0.002 (0.002) | 1.149 | | 1,127.9 | 0.286 |
|  | *-(Freezings)* | Set Up | Tank A | 2.375 (1.367) | 4.979 | | 1,101.7 | 0.028 |
|  |  |  | Tank B | 2.700 (0.145) |  | |  |  |
|  |  | Order |  | 0.034 (0.045) | 0.574 | | 1. 99.1 | 0.451 |
|  |  | Replicate | | -0.072 (0.081) | 0.776 | | 1, 87.6 | 0.381 |
|  |  | Time of Day | | -0.004 (0.002) | 3.170 | | 1, 120.8 | 0.078 |
| FST | *Time in Open* | Set Up | Tank A | -0.267 (1.782) | 0.058 | | 1, 122.9 | 0.810 |
|  |  |  | Tank B | -0.227 (0.167) |  | |  |  |
|  |  | Order |  | -0.052 (0.053) | 0.943 | | 1, 120.8 | 0.333 |
|  |  | Replicate | | 0.025 (0.086) | 0.088 | | 1, 89.8 | 0.767 |
|  |  | Time of Day | | 0.001 (0.003) | 0.041 | | 1, 132.6 | 0.841 |
|  | *-(Food Latency)* | Set Up | Tank A | -0.143 (1.787) | 1.974 | | 1,133 | 0.162 |
|  |  |  | Tank B | -0.393 (0.178) |  | |  |  |
|  |  | Order |  | 0.071 (0.057) | 1.518 | | 1,133 | 0.220 |
|  |  | Replicate | | -0.060 (0.103) | 0.342 | | 1, 133 | 0.560 |
|  |  | Time of Day | | 0.000 (0.003) | 0.008 | | 1, 133 | 0.930 |
|  | *Track Length* | Set Up | Tank A | -3.34 (1.360) | 1.070 | | 1, 130 | 0.303 |
|  |  |  | Tank B | -3.204 (0.131) |  | |  |  |
|  |  | Time in Arena | | 0.159 (0.016) | 99.480 | | 1, 129.4 | 0.000 |
|  |  | Order |  | -0.02 (0.042) | 0.216 | | 1, 129.8 | 0.643 |
|  |  | Replicate | | 0.067 (0.07) | 0.903 | | 1, 91.2 | 0.344 |
|  |  | Time of Day | | 0.003 (0.002) | 2.224 | | 1, 129.3 | 0.138 |
|  | *-(Freezings)* | Set Up | Tank A | -0.078 (1.157) | 4.372 | | 1, 126.7 | 0.039 |
|  |  |  | Tank B | -0.308 (0.110) |  |  | | |
|  |  | Time in Arena | | -0.181 (0.014) | 178 | | 1, 131.9 | 0.000 |
|  |  | Order |  | 0.046 (0.036) | 1.634 | | 1, 126.3 | 0.204 |
|  |  | Replicate | | 0.178 (0.058) | 9.502 | | 1, 90.2 | 0.003 |
|  |  | Time of Day | | 0.002 (0.002) | 0.722 | | 1, 131.7 | 0.397 |

**Tabe S3.** Variance-covariance **ID** matrix estimated from the multivariate model presented in the main text. Variances and covariances with associated standard errors are presented on the diagonal and lower off diagonal respectively.

| **Trait** | *OFT Track Length* | *OFT Area Covered* | *OFT Wall Distance* | *OFT -(Freezings)* | *FST Time in Open* | *FST -(Food Latency)* | *FST Track Length* | *FST -(Freezings)* |
| --- | --- | --- | --- | --- | --- | --- | --- | --- |
| *OFT Track Length* | 0.611 (0.154) | - | - | - | - | - | - | - |
| *OFT Area Covered* | 0.47  (0.13) | 0.488 (0.137) | - | - | - | - | - | - |
| *OFT Wall Distance* | -0.134 (0.087) | 0.017 (0.081) | 0.155 (0.099) | - | - | - | - | - |
| *OFT -(Freezings)* | 0.373 (0.117) | 0.284 (0.103) | -0.12 (0.081) | 0.423 (0.133) | - | - | - | - |
| *FST Time in Open* | -0.239 (0.105) | -0.237 (0.099) | 0.017 (0.079) | -0.149 (0.095) | 0.371 (0.13) | - | - | - |
| *FST -(Food Latency)* | -0.134 (0.084) | -0.073 (0.077) | 0.052 (0.065) | -0.09 (0.077) | 0.081 (0.08) | 0.069 (0.099) | - | - |
| *FST Track Length* | 0.129 (0.073) | 0.047 (0.066) | -0.104 (0.057) | 0.123 (0.067) | -0.046 (0.07) | 0  (0.058) | 0.14 (0.066) | - |
| *FST -(Freezings)* | 0.13  (0.065) | 0.148 (0.061) | -0.004 (0.049) | 0.12 (0.059) | -0.036 (0.061) | 0.029 (0.05) | 0.088 (0.042) | 0.128 (0.051) |

**Table S4.** Estimated effects of experimental tank set up, order, replicate, time of day, sex, and size on OFT and FST behavioural traits. Tank set up was fitted as a two level factor (Tank A, Tank B) and sex as a 3 level factor (female, male, unknown). Tank A, unknown, and unknown:mass are treated as the reference level. Order (1-6) reflects the order shrimp were tested between water changes. Replicate reflects the trial repeat number for the individual (from 1-3). Time of day is recorded as minutes after midnight. Estimates are derived from univariate models with significance tested using conditional F tests.

| **Assay** | **Trait** | **Effect** | **Level** | | **Coefficient (SE)** | | **F,con** | **DF** | **P** |
| --- | --- | --- | --- | --- | --- | --- | --- | --- | --- |
| OFT | *Track Length* | sex | Unknown | 1.114 (1.244) | | | 0.271 | 2,44.7 | 0.764 |
|  |  |  | Female | 1.528 (0.862) | | |  |  |  |
|  |  |  | Male | 1.246 (0.774) | | |  |  |  |
|  |  | Mass |  | 12.387 (6.131) | | | 7.049 | 1, 111.6 | 0.009 |
|  |  | Order |  | 0.004 (0.039) | | | 0.009 | 1, 93.5 | 0.924 |
|  |  | Replicate |  | -0.0854 (0.704) | | | 1.470 | 1, 82.4 | 0.229 |
|  |  | Time of Day | | -0.003 (0.002) | | | 2.453 | 1, 112.3 | 0.120 |
|  |  | Set Up | Tank A | 1.114 (1.244) | | | 0.068 | 1, 93.2 | 0.7944 |
|  |  |  | Tank B | 1.082 (0.126) | | |  |  |  |
|  |  | Sex:Mass | Unknown | 1.114 (1.244) | | | 0.058 | 2, 115.8 | 0.944 |
|  |  |  | Female | -1.883 (10.140) | |  |  | | |
|  |  |  | Male | 1.906(11.608) | | |  |  |  |
|  | *Area Covered* | sex | Unknown | -3.086 (1.273) | | | 0.0717 | 2, 45.8 | 0.931 |
|  |  |  | Female | -1.913 (0.876) | | |  |  |  |
|  |  |  | Male | -1.870 (0.788) | |  |  | | |
|  |  | Mass |  | 18.397 (6.232) | | | 4.608 | 1, 110.4 | 0.034 |
|  |  | Order |  | -0.017 (0.040) | | | 0.179 | 1, 94.9 | 0.673 |
|  |  | Replicate |  | 0.221(0.072) | | | 9.329 | 1, 83.6 | 0.003 |
|  |  | Time of Day | | 0.002 (0.002) | | | 1.047 | 1, 113.5 | 0.308 |
|  |  | Set Up | Tank A | -3.086 (1.273) | | | 15.32 | 1, 94.8 | 0.000 |
|  |  |  | Tank B | -2.581 (0.129) | | |  |  |  |
|  |  | Sex:Mass | Unknown | -3.086 (1.273) | | | 2.073 | 2, 115 | 0.130 |
|  |  |  | Female | -20.171 (19.308) | |  |  | | |
|  |  |  | Male | -22.517 (11.844) | |  |  | | |
|  | *Wall Distance* | sex | Unknown | -0.912 (1.512) | | | 0.251 | 2, 44.8 | 0.779 |
|  |  |  | Female | -0.326 (0.885) | | |  |  |  |
|  |  |  | Male | -0.907 (0.853) | | |  |  |  |
|  |  | Mass |  | 1.983 (6.303) | | | 0.045 | 1, 74.9 | 0.834 |
|  |  | Order |  | 0.0287 (0.051) | | | 0.318 | 1, 110.4 | 0.574 |
|  |  | Replicate |  | -0.220 (0.096) | | | 5.284 | 1, 88.3 | 0.024 |
|  |  | Time of Day | | 0.002 (0.002) | | | 0.906 | 1, 123 | 0.343 |
|  |  | Set Up | Tank A | -0.911 (1.512) | | | 9.116 | 1, 112.5 | 0.003 |
|  |  |  | Tank B | -1.404 (0.163) | | |  |  |  |
|  |  | Sex:Mass | Unknown | -0.911 (1.512) | | | 0.437 | 2, 84.3 | 0.648 |
|  |  |  | Female | -10.417 (10.489) | |  |  | | |
|  |  |  | Male | -1.422 (13.017) | |  |  | | |
|  | *-(Freezings)* | sex | Unknown | 1.586 (1.424) | | | 0.192 | 2, 46.5 | 0.826 |
|  |  |  | Female | 2.735 (0.939) | | |  |  |  |
|  |  |  | Male | 2.852 (0.861) | | |  |  |  |
|  |  | Mass |  | 12.195 (6.697) | | | 0.764 | 1, 101.1 | 0.384 |
|  |  | Order |  | 0.035 (0.046) | | | 0.574 | 1, 99.4 | 0.451 |
|  |  | Replicate |  | -0.074 (0.0829) | | | 0.803 | 1, 85.7 | 0.373 |
|  |  | Time of Day | | -0.004 (0.002) | | | 3.112 | 1, 117.9 | 0.080 |
|  |  | Set Up | Tank A | 1.586 (1.423) | | | 5.044 | 1, 99.6 | 0.027 |
|  |  |  | Tank B | 1.915 (0.147) | | |  |  |  |
|  |  | Sex:Mass | Unknown | 1.586 (1.424) | | | 1.612 | 2, 108.1 | 0.204 |
|  |  |  | Female | -12.163 (11.088) | |  |  | | |
|  |  |  | Male | -33.117 (11.088) | |  |  | | |
| FST | *Time in Open* | sex | Unknown | -0.161 (1.878) | | | 2.076 | 2, 49.1 | 0.136 |
|  |  |  | Female | -0.718 (0.913) | | |  |  |  |
|  |  |  | Male | 1.306 (0.996) | | |  |  |  |
|  |  | Mass |  | -0.608 (5.973) | | | 0.081 | 1, 90.6 | 0.776 |
|  |  | Order |  | -0.054 (0.054) | | | 0.980 | 1, 114.5 | 0.324 |
|  |  | Replicate |  | 0.051 (0.085) | | | 0.362 | 1, 87.9 | 0.549 |
|  |  | Time of Day | | 0.000 (0.003) | | | 0.000 | 1, 126.3 | 0.994 |
|  |  | Set Up | Tank A | -0.161 (1.878) | | | 0.046 | 1, 114.9 | 0.830 |
|  |  |  | Tank B | -0.125 (0.167) | | |  |  |  |
|  |  | Sex:Mass | Unknown | -0.161 (1.878) | | | 1.528 | 2, 95.6 | 0.222 |
|  |  |  | Female | 11.61 (10.402) | | |  |  |  |
|  |  |  | Male | -15.556 (15.261) | |  |  | | |
|  | *-(Food Latency)* | sex | Unknown | -0.827 (1.9136) | | | 1.225 | 2, 127 | 0.297 |
|  |  |  | Female | -2.088 (0.775) | | |  |  |  |
|  |  |  | Male | 0.343 (0.937) | | |  |  |  |
|  |  | Mass |  | -0.939 (5.300) | | | 0.025 | 1, 127 | 0.875 |
|  |  | Order |  | 0.0341 (0.059) | | | 0.329 | 1, 127 | 0.567 |
|  |  | Replicate |  | -0.050 (0.104) | | | 0.228 | 1, 127 | 0.634 |
|  |  | Time of Day | | -3.086 (1.273) | | | 0.395 | 1, 127 | 0.531 |
|  |  | Set Up | Tank A | -0.827 (1.914) | | | 3.805 | 1, 127 | 0.053 |
|  |  |  | Tank B | -1.186 (0.184) | | |  |  |  |
|  |  | Sex:Mass | Unknown | -0.827 (1.914) | | | 2.586 | 2, 127 | 0.079 |
|  |  |  | Female | 9.870 (8.915) | | |  |  |  |
|  |  |  | Male | -24.151 (14.518) | |  |  | | |
|  | *Track Length* | sex | Unknown | -4.143 (1.477) | | | 0.157 | 2, 49.3 | 0.855 |
|  |  |  | Female | -3.449 (0.666) | | |  |  |  |
|  |  |  | Male | -2.915 (0.756) | | |  |  |  |
|  |  | Mass |  | 5.910 (4.441) | | | 0.267 | 1, 75.5 | 0.607 |
|  |  | Time in Arena | | 0.157 (0.017) | | | 89.060 | 1, 125.1 | 0.000 |
|  |  | Order |  | -0.022 (0.044) | | | 0.2473 | 1, 122.5 | 0.620 |
|  |  | Replicate |  | 0.081 (0.072) | | | 1.267 | 1, 89.4 | 0.263 |
|  |  | Time of Day | | 0.004 (0.002) | | | 2.721 | 1, 124.6 | 0.102 |
|  |  | Set Up | Tank A | -4.143 (1.477) | | | 0.951 | 1, 120.9 | 0.332 |
|  |  |  | Tank B | -4.010 (0.136) | |  |  | | |
|  |  | Sex:Mass | Unknown | -4.143 (1.477) | | | 1.465 | 2, 85.5 | 0.237 |
|  |  |  | Female | -11.978 (7.673) | |  |  | | |
|  |  |  | Male | 22.498 (11.652) | |  |  | | |
|  | *-(Freezings)* | sex | Unknown | 0.199 (1.251) | | | 0.818 | 2, 48.8 | 0.447 |
|  |  |  | Female | 0.187 (0.573) | | |  |  |  |
|  |  |  | Male | 0.028 (0.645) | | |  |  |  |
|  |  | Mass |  | 1.587 (3.804) | | | 0.937 | 1, 77.4 | 0.336 |
|  |  | Time in Arena | | -0.187 (0.014) | | | 175.700 | 1, 125.7 | 0.000 |
|  |  | Order |  | 0.056 (0.037) | | | 2.249 | 1, 121.1 | 0.136 |
|  |  | Replicate |  | 0.176 (0.060) | | | 8.657 | 1, 88.4 | 0.004 |
|  |  | Time of Day | | 0.001 (0.002) | | | 0.162 | 1, 125.4 | 0.688 |
|  |  | Set Up | Tank A | 0.199 (1.251) | | | 3.054 | 1, 119.4 | 0.083 |
|  |  |  | Tank B | -0.001 (0.114) | | |  |  |  |
|  |  | Sex:Mass | Unknown | 0.199 (1.251) | | | 0.203 | 2, 87 | 0.817 |
|  |  |  | Female | 2.421 (6.591) | | |  |  |  |
|  |  |  | Male | 6.201 (9.926) | | |  |  |  |

**Tabe S5.** Variance-covariance **ID_Sex:Mass_** matrix estimated from the multivariate model with sex:mass presented in the main text. Variances and covariances with associated standard errors are presented on the diagonal and lower off diagonal respectively.

| **Trait** | *OFT Track Length* | *OFT Area Covered* | *OFT Wall Distance* | *OFT -(Freezings)* | *FST Time in Open* | *FST -(Food Latency)* | *FST Track Length* | *FST -(Freezings)* |
| --- | --- | --- | --- | --- | --- | --- | --- | --- |
| *OFT Track Length* | 0.444 (0.133) | - | - | - | - | - | - | - |
| *OFT Area Covered* | 0.355 (0.116) | 0.425 (0.131) | - | - | - | - | - | - |
| *OFT Wall Distance* | -0.171 (0.088) | -0.012 (0.086) | 0.188 (0.111) | - | - | - | - | - |
| *OFT -(Freezings)* | 0.321 (0.111) | 0.251 (0.103) | -0.168 (0.09) | 0.419 (0.141) | - | - | - | - |
| *FST Time in Open* | -0.34 (0.107) | -0.307 (0.103) | 0.071 (0.086) | -0.199 (0.101) | 0.377 (0.134) | - | - | - |
| *FST -(Food Latency)* | -0.097 (0.085) | -0.042 (0.081) | 0.07 (0.076) | -0.108 (0.086) | 0.095 (0.085) | 0.087 (0.106) | - | - |
| *FST Track Length* | 0.154 (0.077) | 0.058 (0.073) | -0.121 (0.067) | 0.128 (0.076) | -0.096 (0.078) | 0.001 (0.065) | 0.17 (0.078) | - |
| *FST -(Freezings)* | 0.102 (0.064) | 0.132 (0.063) | -0.008 (0.054) | 0.12 (0.064) | -0.062 (0.065) | 0.038 (0.054) | 0.095 (0.047) | 0.13 (0.055) |
